# Taking stock of psychosocial rehabilitation in children and adolescents: a systematic review with meta-analysis

**DOI:** 10.3389/fresc.2025.1568727

**Published:** 2025-11-26

**Authors:** Liesa J. Weiler-Wichtl, Jonathan Fries, Maximilian Hopfgartner, Daniela Feyrer, Kerstin Krottendorfer, Birgit Heller, Caroline Reitbrecht, Ulrike Leiss, Robert Weinzettel

**Affiliations:** 1 KOKON - Rehabilitation for Young People, Rohrbach-Berg, Austria; 2 Department of Pediatrics and Adolescent Medicine, Comprehensive Center for Pediatrics and Comprehensive Cancer Center, Medical University of Vienna, Vienna, Austria; 3 Department of Developmental and Educational Psychology, Faculty of Psychology, University of Vienna, Vienna, Austria

**Keywords:** meta-analysis, psychosocial rehabilitation, systematic review, pediatrics, QOL (quality of life)

## Abstract

**Objective:**

Psychosocial rehabilitation for children and adolescents with physical illnesses remains underdeveloped and poorly defined. This systematic review and meta-analysis aimed to consolidate current research findings.

**Design:**

We searched Medline, PsycINFO, Scopus, Web of Science, and a guideline registry, with the last search conducted on August 30, 2023.

**Subjects/patients:**

Children and adolescents with physical indications.

**Methods:**

Eligible studies reported time-limited rehabilitation programs conducted in dedicated facilities, excluding those focused on psychiatric conditions.

**Results:**

In all, 18 studies were eligible (*N* = 2,933). Meta-analysis (*k* = 4, *i* = 11, *N* = 418) revealed a moderate, statistically significant effect size (*d* = 0.48) for psychosocial rehabilitation in improving quality of life, mood, and anxiety. However, research in this field proved scattered and inconsistent, as few controlled trials were available, and there was little agreement regarding research designs, procedures, and outcome measures.

**Conclusion:**

Psychosocial rehabilitation shows promising effects, but stronger evidence is needed to validate its efficacy. The lack of standardized definitions and procedures hinders progress. Future research should focus on randomized controlled trials and larger samples to optimize rehabilitation practices and improve outcomes for young patients with somatic conditions, resulting in evidence-based guidelines.

**Systematic Review Registration:**

https://doi.org/10.17605/OSF.IO/AM2Z9

## Introduction

Psychosocial rehabilitation involves treating patients outside acute care settings within a defined timeframe, emphasizing interventions like clinical psychology, psychotherapy, psychosocial counseling, art therapy, or pedagogic methods to enhance overall and condition-specific well-being and prepare patients to resume their daily activities. This approach aligns with the World Health Organization's mental health action plan (1). In recent years, the concept has garnered substantial attention within the scientific community, as evidenced by a PubMed search of the term “psychosocial rehabilitation,” which returned over 1,000 results, with 80% of these publications appearing since 2000. Delving into the literature on rehabilitation, it becomes clear that the term “rehabilitation” encompasses a broad spectrum regarding time point, duration, treatment methods, and professionals involved (2, 3). However, psychosocial rehabilitation—particularly for children and adolescents—currently lacks clear definitions and standardized approaches (4, 5). This ambiguity exposes the field to so-called jingle-jangle fallacies: some studies may refer to “psychosocial rehabilitation” but describe fundamentally different approaches (jingle), while others may employ fairly similar approaches but use different names (jangle) (6). This can lead to fragmented, inconsistent, and irreproducible research, ultimately resulting in ambiguous treatment recommendations for clinical practice. Psychosocial rehabilitation is defined in this review as a time-limited intervention conducted outside of acute care settings. It is focused on enhancing well-being and functional outcomes through multidisciplinary approaches such as psychotherapy, counseling, and pedagogic methods (7). While general psychosocial support or counseling are aimed at addressing acute psychological needs (8), psychosocial rehabilitation adopts a holistic approach. It integrates psychological, social, and developmental aspects with the goal of enhancing the young patients’ independence and alleviating broader psychosocial stressors. This conceptualization aligns with the biopsychosocial model (9) and emphasizes the diverse needs of children and adolescents with somatic conditions. Chronic physical illnesses often have substantial psychosomatic implications, as psychological factors such as stress, anxiety, and depression can exacerbate physical symptoms, while the illness itself may lead to emotional and social challenges (10). Moreover, the impact of illness extends to the young patients’ families, where parental stress, sibling dynamics, and overall family functioning may influence a child's rehabilitation outcomes (11). Psychosocial factors also play a critical role in treatment adherence and coping, as emotional well-being and social support are essential for fostering resilience and providing sustainable health improvements (12, 13).

In Austria, for instance, existing psychosocial rehabilitation practices follow the guidance of service portfolios administered by the Austrian health insurance (Sozialversicherung), and there are four Austrian facilities offering psychosocial and mental health rehabilitation (14–16). However, it is to date unclear if such models adequately meet the specific needs of pediatric populations, which require consideration of their unique developmental stages, integration within family structures, and the broader process of maturing into adulthood (17–22). One central question in this field is determining the appropriate level of involvement of parents and families in the rehabilitation process. While the primary focus remains on the patients, it is crucial to consider parental perceptions and the influence they exert on therapy outcomes. Child- and adolescent-oriented rehabilitation poses specific challenges, especially when parents act as mere companions, underscoring the need for integrated systems that address both the needs of the child and the high burden on parents (23, 24). Another important aspect is the context-dependent treatment of mental health and psychosocial issues. This discussion focuses specifically on non-psychiatric aspects of mental health, defining it in terms of well-being, empowerment, and health literacy (25–28).

The diverse use and wide-ranging applications of psychosocial rehabilitation underscore the crucial need for standardized yet individualized guidelines in the field. Clarifying what constitutes rehabilitation, distinguishing it from primary care and aftercare, and understanding the specific indications are necessary to improve outcomes. The success of rehabilitation depends on the collaboration of multiple disciplines, necessitating standardized approaches that allow for comparability and precise assessment of therapeutic goals. The involvement of diverse professional groups—such as psychologists, psychotherapists, art and music therapists, social educators, and social workers—is understood to be essential for effective psychosocial rehabilitation. An interdisciplinary framework in which these professionals collaborate closely within an integrated care system (29) encompassing medical, physical, and psychosocial components is vital (30, 31).

While psychosocial elements are not implemented ubiquitously across indications, they are applied routinely in the treatment of psychiatric conditions such as depression or anxiety disorders (32, 33). Consequently, the psychosocial aspects in the rehabilitative treatment of psychiatric conditions are defined in specific guidelines (34, 35). Conversely, it is currently unknown how effective psychosocial rehabilitation programs are when the indications are not primarily psychiatric (i.e., somatic conditions) and which factors contribute to their effectiveness.

It is reasonable to assume that additional patient populations beyond those with somatic conditions could benefit from psychosocial rehabilitation, particularly when considering that health and illness are inherently biopsychosocial phenomena. The biopsychosocial model acknowledges the complex interaction between biological, psychological, and social factors in both the onset and management of illness. Consequently, psychosocial rehabilitation could play a crucial role in addressing the mental and social components of health, which are often underemphasized in traditional medical care (31, 36–38).

Thus, the goal of the present review was to assess the present state of research in psychosocial rehabilitation in children and adolescents with somatic conditions. Specifically, we examined the components of existing psychosocial rehabilitation programs, the methods used to evaluate them, and their effectiveness. To achieve this, we conducted a systematic review of the relevant literature and their methodological characteristics, complemented by a multilevel meta-analysis to estimate the overall effectiveness of these rehabilitation programs. In this review, we use the term “mental health” as a broader concept encompassing overall emotional and psychological well-being. We use the term “rehabilitation program” as an umbrella term for all time-limited inpatient interventions, which may include, among others, interventions such as clinical psychology, psychotherapy, psychosocial counseling, art therapy, or pedagogic methods.

## Methods

In the present study, we followed the Preferred Reporting Items for Systematic Reviews and Meta-Analyses guidelines [PRISMA; (39)]; the hypotheses, literature search strategy, and analysis plans were pre-registered using the PROSPERO framework [(40); registration available at https://doi.org/10.17605/OSF.IO/AM2Z9].

### Literature search and selection criteria

We conducted a systematic literature search of the databases Scopus, Medline, Web of Science, PsycINFO, and AWMF (guideline registry). The search started on June 1st, 2023, and the last search occurred on August 30th, 2023. The exact search queries for the respective databases are available in the project registry (https://doi.org/10.17605/OSF.IO/AM2Z9). In addition to our pre-registered search strategy, we assessed the cited references of one systematic review (41) to identify further relevant articles.

Because we aimed to provide a comprehensive view of the literature on psychosocial rehabilitation in children and adolescents, we included all available empirical articles. However, to be included, studies had to meet several pre-defined criteria. First, we required the patients (or, in the case of non-empirical works, the patient populations to which the article was targeted) to be younger than 18 years. Second, subjects had to be enrolled in an in- or outpatient rehabilitation program (or the article had to describe such a program) in a specialized institution. Third, the rehabilitation program had to include substantial psychological, psychosocial, social, mental health, pedagogical, or psychological assessment elements. Fourth, the rehabilitation program had to occur in a limited time window. We excluded articles that referred to acute or primary care programs because their focus was not on rehabilitation. Notably, as we aimed to describe psychosocial rehabilitation in children and adolescents with somatic conditions, we excluded all articles targeted at patient populations with psychiatric indications. We also excluded literature reviews and theoretical contributions. Finally, we excluded papers that focused on a target population other than the patients themselves (e.g., therapists or health services).

### Data extraction

Two coders (JF and MH) coded the included primary studies in March 2024. Both coders assessed all studies. They initially agreed upon 96 percent of the coding decisions. Discrepancies were resolved by consulting with a third researcher (LWW). Wherever available and applicable, we extracted the following study-level characteristics: (1) publication year, (2) authors, (3) title, (4) journal, (5) DOI, (6) total sample size, (7) sample size female, (8) sample size male, (9) mean age at admission, (10) profession (responsible for psychosocial aspects of the program), (11) illness/disorder, (12) parent involvement, (13) type of intervention, (14) duration of intervention, (15) outcomes, (16) concept of rehabilitation, (17) control group, (18) method(s) used to assess outcomes, (19) time of measurement, (20 & 21) mean and *SD* of assessed outcome pre-intervention, (22 & 23) mean and *SD* of assessed outcome post-intervention, and (24) coding date.

We performed a formal analysis of primary study quality using the Newcastle-Ottawa scale, adapted for nonrandomized studies (42, 43); the manual for study quality coding is provided in Supplementary Section 1. In addition, we reported study characteristics and potential sources of bias, consistent with well-established recommendations (44).

### Meta-analysis

In addition to a comprehensive overview of the available literature, we aimed to meta-analytically estimate the efficacy of psychosocial rehabilitation in children and adolescents. However, because we included a wide range of article types, only a small subset of articles was available for quantitative synthesis. To be included in our meta-analysis, studies had to report numeric results (*M* and *SD*) of assessments of either quality of life (QoL) or mental or physical health assessments before and after the intervention. Following common recommendations, we synthesized studies even if they reported different outcomes, given that they had applied the same designs (45). From these results, we calculated standardized mean differences (Cohen *d*) for paired samples (45, 46). In cases where a negative effect size (i.e., a reduction of the measured outcome) represented a beneficial treatment effect, we inverted the effect size to match the other outcomes (i.e., for depression). Thus, positive effects generally represent beneficial treatment effects in the current meta-analysis. Further details on effect size calculations are documented in Supplementary Section 2.

Because some studies reported multiple outcomes, we computed multilevel random-effects models (REM) with restricted maximum-likelihood estimation and inverted variance weights to synthesize the effect sizes (47). Henceforth, we refer to reports (that may contain multiple effect sizes) with *k*; we refer to effect sizes with *i*. We estimated the variance components (i.e., heterogeneity between studies and within studies) for the different levels following standard practice (48).

We investigated potential confounding effects of publication bias using two methods suitable for multilevel meta-analyses: Egger's multilevel meta-regression and Egger's regression with sandwich estimators (49).

Effect sizes were interpreted according to well-established guidelines (50), discriminating between very small (*d* < 0.2), small (0.2 ≤ *d* < 0.41), moderate (0.41 ≤ *d* < 0.63), and large effects (*d* ≥ 0.63).

For quantitative analyses, we used R 4.4.0 (51). For meta-analytic calculations, we used the R package *metafor* (47). Figures were created using the R packages *ggplot2* (52) and *metaviz* (53). The analysis code is available in Supplementary Section 3.

## Results

### Study selection

**Figure 1:**
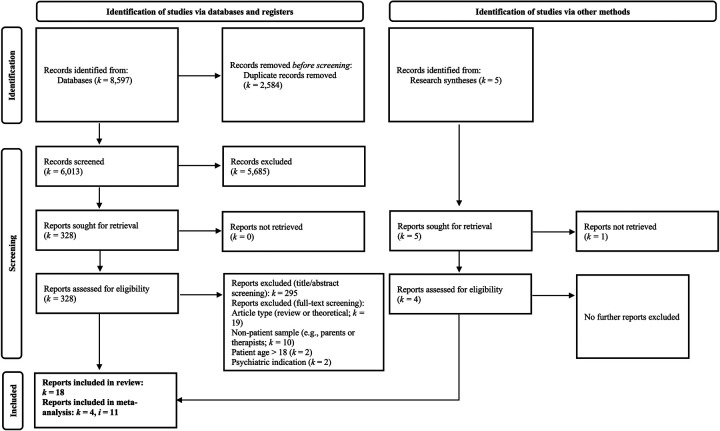
PRISMA flow chart.

In all, we identified 8,597 potentially includable research items. After title and abstract screening, deduplication, and full-text screening, 49 articles remained. From these articles, we excluded articles that did not report empirical results (*k* = 10) and literature reviews (*k* = 9). Further, we excluded articles that had a sample population other than the patients themselves (*k* = 10). We also excluded *k* = 2 articles that investigated patients with psychiatric conditions, as well as *k* = 2 articles that involved patients older than 18 years of age and *k* = 1 article that reported rehabilitation outcomes with unclear psychosocial components. In addition to studies identified through forward database search, we included *k* = 3 reports (54–56) discovered in the reference list of a systematic review (41). Our final literature selection comprised *k* = 18 empirical articles (*N* = 2,933) (54–71). See Figure 1 for a PRISMA flow chart of the literature selection process. Table 1 shows detailed study characteristics with references.

**Table 1:** Study characteristics.

Short reference	*N* (female)	Mean age	Profession	Study design	Target condition	Parent involvement	Duration (days)	Outcomes	Setting	Country
Banez et al. (2014) (55)	173 (127)	15.00	Multidisciplinary	Retrospective chart review	Chronic pain	Children separated from parents	21.00	Hospital	United States	
**Benore et al. (2015) (57)**	**119** (**91)**	**15**.**40**	**Psychology**	**Single-arm study with two timepoints**	**Chronic pain**	**Unclear**		**Anxiety, pain catastrophizing, qol, pain-related functioning**	**Hospital/day-clinic**	**United States**
**Däggelmann et al. (2017) (58)**	**22** (**10)**	**10**.**36**	**Multidisciplinary**	**Two-arm nonrandomized study**	**Cancer**	**Family-oriented intervention**		**Motor performance, qol, fatigue**	**Hospital**	**Germany**
Ennis et al. (2014) (61)	20 (7)		Medicine	Retrospective chart review	Brain injury	Medium, family-level is included		Quality-of-care	Hospital	United States
Goldbeck et al. (2011) (54)	302 (128)	8.70	Multidisciplinary	Two-arm nonrandomized study	Cancer	Family-oriented intervention	30.00	Health related qol	Hospital	Germany
Hampel et al. (2020) (62)	142 (87)		Psychology	Survey	Obesity	Medium; parents were surveyed		Mental problems	Hospital	Germany
Kasia et al. (2021) (63)	142 (103)	13.78	Multidisciplinary	Three-arm nonrandomized study	Functional neurological disorder and mental health comorbidities	Family-oriented intervention	14.00	Functional status	Hospital	Australia
**Maynard et al. (2010) (59)**	**41** (**30)**	**13**.**80**	**Psychology**	**Retrospective chart review**	**Physical and intellectual disabilities**	**Caregiver participated in treatment**	**27**.**00**	**Medication usage**	**Hospital**	**United States**
Nelson et al. (2019) (71)	305 (251)	14.38	Multidisciplinary	Single-arm study with two timepoints	Chronic pain	Family-oriented intervention	25.50	Somatic symptoms, depressive symptoms, anxiety symptoms, pain-related experience, functional status	Day-clinic	United States
Pakhomova et al. (2022) (64)	50 (27)	8.50	Psychology/pedagogy	Rct	Physical and intellectual disabilities	Unclear		Well-being, activity, and mood	Unclear	Ukraine
Papp et al. (2021) (65)	60 (41)		Psychology	Retrospective online survey	Various	None		Health acceptance	Camp	Hungary
**Riedl et al. (2022) (60)**	**236** (**97)**	**11**.**00**	**Psychology**	**Single-arm study with two timepoints**	**Cancer**	**Family-oriented intervention**		**Health related qol**	**Hospital**	**Austria**
Risko (2019) (66)	6 (5)	15.70	Psychology	Interviews with patients	Chronic pain	Parents not interviewed, but metioned often		Experiences in rehabiliation	Hospital	United States
Schiel et al. (2017) (56)	901 (468)	11.50	Multidisciplinary	One-arm nonrandomized study	Diabetes	In younger children, parents participated	30.00	Blood glucose and other diabetes-related blood markers, adherence to self-administered blood glucose measurement, bmi	Hospital	Germany
Singer and Drotar (1989) (67)	127 (NA)		Psychology	Analysis of referrals to psychological consultation service	Chronical illness	Parents mentioned			Hospital	United States
Svatenkova (2016) (68)	77 (NA)		Psychology	Not defined	None			Camp	Ukraine	
Xodo (2020) (69)	NA (NA)		Psychology	Presentation of intervention	Asthma	Parents involved in intervention			Hospital	Italy
Zakrepina et al. (2020) (70)	210 (NA)		Rehabilitation medicine	Survey	Brain injury	None		Mental activity	Hospital	Russia

QoL, quality of life; RCT, randomized controlled trial. Studies set in boldface were included in our meta-analysis.

### Study characteristics

All included articles were studies reporting investigations of psychosocial rehabilitation programs in children and adolescents with somatic indications. Most articles were published after 2010 (54–66, 68–71), whereas only one article was published earlier (67).

On average, participants were *M* = 12.56 years old (*SD* = 2.65); note, however, that *k* = 7 studies did not report sufficient statistical information to compute the mean age (61, 62, 65, 67–70). Between studies, sample sizes varied substantially (*M* = 172.53, *Mdn* = 127, *SD* = 209.91).

**Figure 2:**
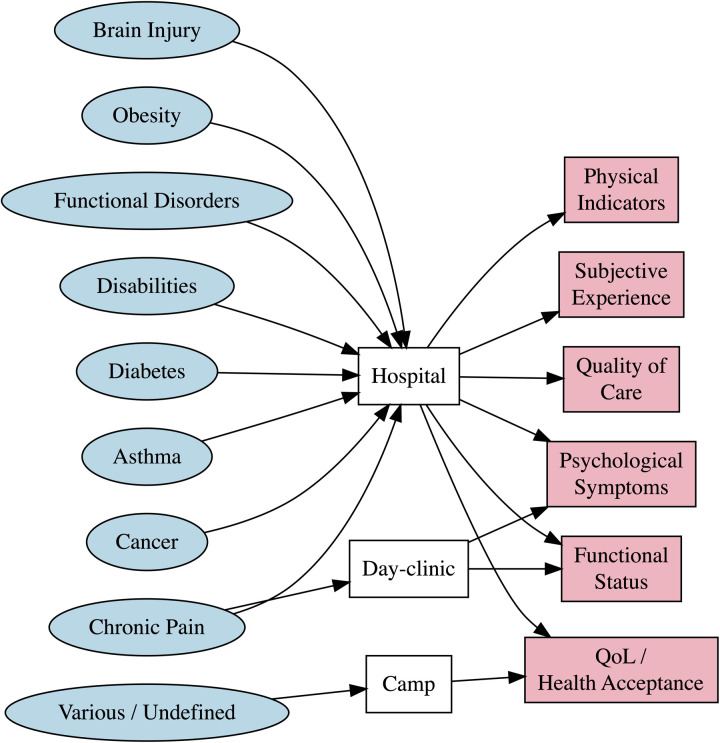
Outcome measures by setting and target conditions.

However, the included reports were heterogeneous regarding their patient populations. Chronic pain patients were the target population of *k* = 4 studies (55, 57, 66, 71), followed by cancer and leukemia (*k* = 3) (54, 58, 60), physical and intellectual disabilities (*k* = 2) (59, 64), brain injuries (*k* = 2) (61, 72), various conditions (*k* = 1) (65), obesity (*k* = 1) (62), asthma (*k* = 1) (69), diabetes (*k* = 1) (56), functional neurological disorder (*k* = 1) (63), and chronic illness (*k* = 1) (67), while *k* = 1 study did not report a clear definition of their patient population (68). Thus, the examined health conditions varied substantially across studies. Figure 2 shows a diagram of target conditions, settings, and outcome measures.

We encountered a wide range of study designs within the literature. Most frequently, studies used a single-arm nonrandomized design (*k* = 4) (56, 66, 67, 69). Single-arm nonrandomized studies with pre- and post-assessments of outcomes were applied by *k* = 3 studies (57, 60, 71), as well as retrospective chart reviews (*k* = 3) (55, 59, 61), and surveys (*k* = 3) (62, 65, 70), followed by two-arm nonrandomized studies (*k* = 2) (54, 58) and *k* = 1 three-arm nonrandomized study (63), while *k* = 1 study reported insufficient information on the trial's design (68). We only encountered *k* = 1 randomized controlled trial (RCT) (64). Only a small fraction of studies (*k* = 2) (54, 64) included a control group, whereas the remainder of studies (*k* = 16) did not (55–63, 65–71). This was also reflected in the study quality, which we assessed using an adapted version of the Newcastle-Ottawa scale. On average, studies showed mediocre quality (*M* = 5.39, *SD* = 1.91; the highest possible score was 8); more details on the quality of the included studies can be found in Supplementary Section 1 and Supplementary Table 1.

Most rehabilitation programs were carried out in strictly inpatient, institutional environments (*k* = 10) (54, 56, 58–62, 64, 67, 70), whereas *k* = 5 rehabilitation programs additionally included outpatient elements (55, 57, 63, 66, 71). Rehabilitation camps were the site for *k* = 2 studies (65, 68). Further, *k* = 1 study reported inpatient rehabilitation with unclear outpatient elements (69). On average, the rehabilitation programs lasted for approximately three weeks (*M* = 24.58, *SD* = 6.17).

Across the examined psychosocial rehabilitation programs, psychologists were the most prevalent professional group responsible for the psychosocial aspects of rehabilitation (*k* = 9) (57, 59, 60, 62, 65–69). In *k* = 6 programs, the psychosocial personnel comprised a multidisciplinary team of more than two professional groups (54–56, 58, 71). In *k* = 1 study, the psychosocial aspects were covered by a combination of psychologists and pedagogues (64), while *k* = 2 described their psychosocial personnel were described as rehabilitation medical professionals (61, 72).

Generally, parental involvement in the rehabilitation programs was fairly prevalent. Parents were involved in *k* = 12 studies (54, 58–63, 66–69, 71, 73), albeit to varying degrees. Only *k* = 3 studies reported no parental involvement whatsoever (65, 68, 70), and in *k* = 1 study (55), children were separated from their parents. In *k* = 2 reports, parental involvement was not specified (57, 64).

Nearly all studies relied heavily on self-report measures, as *k* = 16 articles stated that the psychsocial outcomes were assessed using self-report measures (54–66, 68, 70, 71); for the remaining *k* = 2 studies, the mode of measurement was unclear (67, 69). However, there was little agreement between the studies regarding which outcomes should be assessed. The most frequently used outcome was quality of life (QoL), which was assessed in *k* = 4 studies (54, 55, 58, 60). Depressive symptoms and mood were assessed in *k* = 2 studies (64, 71), and anxiety was also assessed in *k* = 2 studies (57, 71). The remainder of the outcome measures were quite heterogeneous; details are displayed in Table 1. On average, post-intervention outcomes were measured at a follow-up time of *M* = 32.18 days post-admission (*SD* = 10.64); however, *k* = 13 studies reported insufficient data on follow-up time (57, 58, 60–70).

### Meta-analysis

In addition to a descriptive account of study characteristics, we aimed to quantitatively estimate the efficacy of the included rehabilitation programs using meta-analysis. However, due to data unavailability and differences in study designs, only a small subset of articles that reported evaluations of the efficacy of psychosocial rehabilitation programs in children and adolescents with somatic conditions was available for quantitative synthesis (*k* = 4, *i* = 11) (57–60). Our meta-analytic dataset contained data on 418 individual patients. The studies included in our meta-analysis are summarized in Table 1.

Notably, almost all effect sizes indicated beneficial treatment effects. However, one effect size showed an inconsistent direction compared to the other effects from the same article (57). This effect size indicated a decrease in psychosocial QoL over the course of the intervention, whereas physical QoL showed a positive treatment response (57).

**Figure 3:**
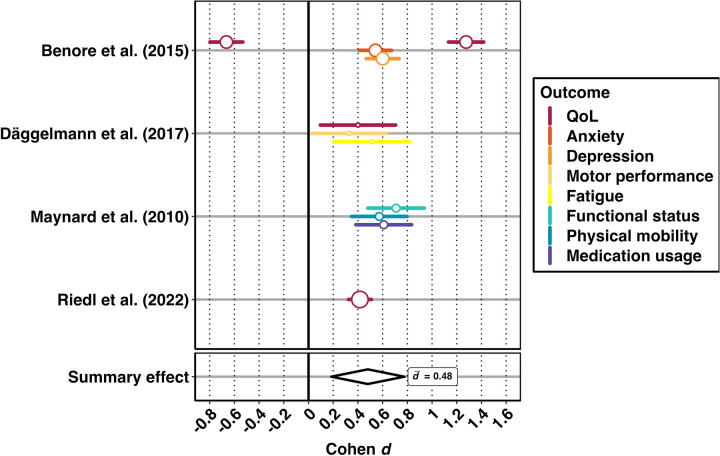
Forest plot of the meta-analysis of intervention studies. The *x*-axis indicates Cohen *d*. Larger symbol sizes indicate larger samples. The *y*-axis shows individual studies. The circles with horizontal bars correspond to effect sizes and their confidence intervals. Colors indicate different outcomes. The vertical reference line demarcates the null effect (*d* = 0.00). The center of the bottom diamond indicates the overall summary effect, while its width shows the corresponding confidence interval.

Our multilevel REM yielded a statistically significant, moderate effect size across a wide range of outcomes [*d* = 0.48, 95% CI (0.18, 0.78), *p* = .001, *I*^2^ = 88.31, *σ*^2^_between_ = 0.01, *σ*^2^_within_ = 0.21, *i* = 11]. The positive summary effect indicates that, on average, participants reported improved levels in the respective outcomes after the intervention compared to before the intervention. Notably, the effect sizes showed a clear pattern of beneficial treatment responses, irrespective of the marked variety of included outcomes. Effect sizes displayed substantial heterogeneity, with most of the heterogeneity attributable to variability within studies. In other words, effect sizes varied more markedly between the effect sizes reported in a study than between the effect sizes reported in different studies. Figure 3 shows a forest plot of the meta-analytic REM.

We conducted a subgroup analysis, including only effect studies examining QoL. The model yielded a small, statistically insignificant effect size [*d* = 0.36, 95% CI (−0.44; 1.15), *p* = .38, *I*^2^ = 96.91, *σ*^2^_between_ = < 0.01, *σ*^2^_within_ = 0.63, *i* = 4, *n* = 377]. However, confidence intervals were large, as statistical power was limited due to the low study count. The number of studies for the remaining outcomes was insufficient for further subgroup analyses.

Our multilevel publication bias methods yielded no indication of publication bias, as Egger's multilevel meta-regression (*z* = 0.93, *p* = .35) and Egger's regression with robust variance estimation (*t* = 3.56, *p* = .11) yielded non-significant results. This means that selective reporting was unlikely to have influenced the results of the meta-analysis. However, these results need to be interpreted cautiously, as statistical power was limited (49).

## Discussion

Here, we present the first systematic account of psychosocial rehabilitation in children and adolescents with somatic conditions. Our findings provide tentative support for the effectiveness of such programs, as we uncovered a moderate, statistically significant summary effect size in our meta-analysis of evaluation studies (*d* = 0.48). The effect proved robust across a wide range of medical indications, such as chronic pain, cancer, or diabetes, as well as different outcomes, including QoL, mood, anxiety, and condition-specific indicators. This indicates that after rehabilitation, the young patients fared better in various aspects of their lives than before, irrespective of their medical conditions. However, it is important to note the preliminary nature of these meta-analytic findings, as the study pool was quite small.

At the outset of the present study, we asked two fundamental questions: First, what is psychosocial rehabilitation in children and adolescents with somatic conditions? And second, what makes good psychosocial rehabilitation in children and adolescents with somatic conditions? Based on our findings, we can answer neither of them with confidence. Our data reveal that research on psychosocial rehabilitation in children and adolescents with somatic conditions remains a scarce and scattered landscape. Despite our efforts to cast a wide net in our literature search, the number of available studies was very small, as only 18 reports matched our criteria (54–71). Even smaller was the number of targeted investigations on the effectiveness of such rehabilitation programs that were includable in our meta-analysis (*k* = 4) (57–60), limiting its generalizability.

This leads us to the conclusion that there is a pressing need for the development, validation, and implementation of psychosocial rehabilitation programs. Several areas with known psychosocial burdens, which have significant potential for benefiting from rehabilitation, remain underexplored in the literature. For instance, pain management—encompassing conditions such as somatoform pain, migraines, headaches, and abdominal pain—remains underrepresented despite its complexity and prevalence. A dedicated rehabilitation approach for these conditions could provide comprehensive care, integrating medical treatment with psychological, pedagogical, and social support and offering a holistic approach to pain relief that is often absent in standard care. Another critical yet insufficiently addressed area is the transition to adulthood for adolescents with chronic illnesses. This phase presents unique challenges, such as managing increased responsibilities while coping with persistent health issues. A specialized rehabilitation program tailored to these adolescents could offer the necessary support to navigate this transition, ensuring continuity of care and helping them build resilience as they enter adulthood. The psychosocial aspect of developmental disorders, including those associated with syndromes and genetic conditions, as well as speech and language delays, also merits further attention. While these conditions are recognized, the potential for rehabilitation to address the associated cognitive, social, and emotional difficulties is not fully explored.

Additionally, the psychosocial stressors experienced by children following parental divorce, separation, or loss, and, in addition, psychosocial resources are areas that merit exploration within a rehabilitation context. These experiences can deeply affect a child's emotional well-being, and a structured rehabilitation program could offer essential support. Moreover, many aspects of daily life can be burdensome: school stress, climate change, the pandemic, and the challenges of growing up. A particularly difficult experience is dealing with a family member's illness, whether it involves a parent or sibling, and whether the illness is physical, like cancer, or a mental health condition. Such an illness can disrupt daily life and emotionally strain the entire family. Early psychosocial support can potentially help manage these challenges and maintain one's own mental well-being (74). Assessing whether psychosocial rehabilitation could be an effective intervention for these issues is an important area for future research. This emphasizes the need for distinguished outcome measures to evaluate the progress and precise and realistic rehab goals.

Still, our systematic review of the literature revealed some consistent findings. Typically, patients were around 13 years of age when admitted to rehabilitation, and their stay had an average duration of roughly three weeks. Most rehabilitation programs were situated in inpatient, institutional settings. Notably, there was some agreement across studies that psychologists should be part of such rehabilitation programs (57, 59, 60, 62, 65–69), while there was less agreement regarding the involvement of other professional groups, such as pedagogues or occupational therapy (64, 71). Similarly, most studies agreed that parents should have at least some role in the rehabilitation of their children (54, 58–63, 66, 67, 69, 71, 73). Importantly, these commonalities only reflect how psychosocial rehabilitation in children and adolescents with somatic conditions is currently implemented in clinical practice. This, however, does not necessarily indicate what and how it ought to be implemented. It will be essential to define the key therapeutic factors (e.g., shared rehabilitation cycles, group compositions, age and developmental stages, thematic focus, group sizes, and specific interventions and their interplay). Moreover, it is crucial to establish relevant key performance indicators (KPIs) to demonstrate the effectiveness of these approaches (75). These KPIs must extend beyond merely assessing quality of life. They should directly address the core objectives of rehabilitation as outlined by the WHO (76): enhancing health literacy, ensuring the transfer and sustainability of interventions in daily life, fostering the development of independence, and facilitating reintegration into an (age- and developmentally-) appropriate daily routine. Establishing such KPIs will not only provide a concrete measure of rehabilitation outcomes but also ensure that the program's effectiveness is aligned with its fundamental goals. The logical outcome should be the development of a guideline for psychosocial rehabilitation, which incorporates future relevant findings and includes a consensus-based approach, including crucial healthcare professionals as well as the patient groups themselves (77, 78).

The scattered nature of the literature was also reflected in the characteristics of applied research designs. We encountered only one randomized controlled trial (64), and in total, only two studies included a control group (54, 64). Other studies implemented a wide range of nonrandomized, uncontrolled research designs (55–63, 65–71). This is unfortunate because, in the absence of a group of either healthy controls or a waiting group that does not receive a rehabilitative intervention, we cannot rule out that the effects are simply caused by the passage of time. A genuine treatment effect is only indicated if the treatment group shows a larger improvement compared to the control group (79). Another problematic issue is the partial lack of statistical power, as five studies had sample sizes of 50 participants or fewer (80).

We encountered a wide range of outcome measures, with QoL being included most frequently (54, 55, 58, 60). Quality of life (QoL) in the context of pediatric psychology is often defined as a child's subjective perception of well-being across multiple life domains, including physical health, emotional functioning, family and peer relationships, and participation in age-appropriate activities (81, 82). Our meta-analytic subgroup analysis for QoL yielded a small positive treatment effect, albeit non-significant, due to a probable lack of statistical power. One study investigated different subcategories of QoL and found that while physical QoL improved, psychosocial QoL was negatively affected (57). A likely explanation is that being isolated from one's friends in an inpatient setting could (transiently) leave social needs unfulfilled (57). Unfortunately, only one study investigated QoL on a subscale level, and future studies should make efforts to investigate QoL in more detail, as this could generate valuable cues about possible improvements to existing rehabilitation programs. Apart from QoL, few outcome measures were applied consistently across studies. On the one hand, this is hardly surprising, as the patients’ indications for rehabilitation varied greatly, and different health conditions require different outcome measures. On the other hand, however, it is important to determine a set of default outcomes and measures to establish a condition-independent common core of psychosocial rehabilitation standards for children and adolescents with somatic conditions. This is only possible if researchers uncover which outcomes are affected in which ways by psychosocial rehabilitation. Based on our findings, we cannot draw such conclusions, as the number of studies for each outcome measure was simply too small to conduct subgroup or moderator analyses.

It is important to identify key focus areas particularly suited for psychosocial rehabilitation in pediatric and chronic conditions. Psychosocial stressors experienced by children and their siblings, particularly in the context of pediatric or chronic illnesses, emerge as a critical area of concern. This aspect encompasses the wide-ranging emotional and psychological challenges that these children face, often impacting their overall well-being and development (19). Further elaboration on this topic could significantly enhance our understanding, ideally resulting in the development of standardized programs tailored to various somatic conditions, considering persistent psychosocial stressors. This should involve incorporating frameworks for the structure and components of rehabilitation competency (83). Moreover, frameworks such as the Consolidated Framework for Implementation Research are suggested for successful implementation (84) alongside participative developmental processes (85). A subsequent step is to establish standards across the entire rehabilitation process—from admission and initial consultation to individualized treatment planning, therapy evaluation, discharge, and follow-up care. Effective interface management and an understanding of the significance of pre-admission processes are necessary for successful rehabilitation, particularly for children, who are often dependent on systemic support. This requires the establishment of structures that accommodate family-oriented approaches or legal frameworks, such as parental leave entitlements. Furthermore, it is crucial to encourage a broader societal recognition of the importance of rehabilitation, highlighting that it is not a luxury but an essential component of healthcare. All mentioned aspects should be consolidated into a comprehensive guideline to ensure the standardization of practices and facilitate the consistent implementation of evidence-based strategies to follow good clinical practice (86).

In response to the heterogeneity observed in existing psychosocial rehabilitation programs for children and adolescents with somatic conditions, we propose the development of a core outcome set to guide future research and evaluation efforts. This set should include standardized measures of psychosocial functioning, emotional well-being, participation, and family burden. Additionally, while program structures varied considerably across studies, our review identified recurring elements that may serve as the basis for future model development and quality benchmarks. These include a time-limited duration (typically 3–5 weeks), the integration of both individual and group-based interventions, and a structured daily routine that supports therapeutic engagement. Interventions should be tailored in intensity and content to the developmental needs of the target population and address goals such as daily activation, emotional regulation, social re-engagement, and disease management. A comprehensive psychosocial rehabilitation model should also incorporate standardized psychosocial assessments, ensure meaningful parental involvement, and address relevant domains such as emotional regulation, body image, family dynamics, and resilience. Interdisciplinary teams—including psychosocial, medical, and therapeutic professionals—are essential for delivering holistic care. Finally, suitable evaluation strategies, including pre-post and follow-up assessments, as well as mechanisms for transition planning and long-term monitoring, are critical for sustaining outcomes and enabling continuous improvement.

## Limitations

We have already discussed some of the limitations we encountered in the current systematic review. However, certain limitations are inherent in every literature review (87). We made substantial efforts to uncover all available articles that met our criteria. These criteria were fairly narrow, as we only included rehabilitation programs that were time-limited, carried out in an institutional setting, and were not specifically targeted to psychiatric indications. Thus, we excluded many articles that investigated other forms of rehabilitation. However, our aim was to take stock of this arguably underdeveloped and ill-defined subdiscipline of rehabilitation research. Despite our comprehensive literature search, it is nonetheless possible that we missed relevant articles.

Our meta-analysis included four articles (reporting 11 effect sizes) selected based on strict inclusion criteria, focusing on psychosocial rehabilitation programs for children and adolescents with somatic conditions and providing sufficient data for effect size calculation. While the small number of studies is a limitation, pooling them was justified by their shared focus and comparable outcome measures, such as quality of life and psychological well-being. This allowed us to provide a preliminary estimate of effectiveness, with a moderate summary effect size (*d* = 0.48). However, the limited evidence base restricts the generalizability of these findings, which should be interpreted with caution. We explored heterogeneity using the *I*^2^-statistic and by calculating the variance components associated with each level of analysis (48), which indicated low-to-moderate variability, but the small sample size precluded meaningful moderator and sensitivity analyses.

None of the studies in our meta-analysis applied a controlled design, limiting the generalizability of the results. We thus encourage researchers to implement control groups into their research designs. Further, we were unable to compute moderator analyses to evaluate factors that influence the effectiveness of the rehabilitative treatment. As the field of research on psychosocial rehabilitation in children and adolescents with somatic conditions shows recent growth, we encourage researchers to reproduce and extend our meta-analysis, including additional studies published in the future.

In recent years, inclusion and equity have been increasingly discussed in rehabilitation research (88–90). However, in the literature we reviewed here, only few studies have focused meaningfully on these topics. For instance, some studies highlight barriers to access, such as geographic and financial limitations, which restrict participation in intensive rehabilitation programs for certain groups, including those with adverse childhood experiences (ACEs) or lower socioeconomic status (71). Additionally, while some articles mention cultural awareness and the need for culture-specific advocacy resources (61), adherence to these practices was low across facilities, and implementation was inconsistent, reflecting a gap in addressing the needs of diverse populations. Overall, while the need for equity-oriented practices is acknowledged in some articles, their implementation and reporting remain insufficient—a gap that should be addressed in future work.

In conclusion, in the current systematic review of psychosocial rehabilitation in children and adolescents with somatic conditions, we encountered a highly heterogeneous field with few common denominators. We found preliminary evidence that such rehabilitation programs can have a positive impact on a variety of outcomes in a multitude of health conditions. However, too few high-quality, targeted investigations on the effectiveness of such rehabilitation programs are currently available. Investigating psychosocial rehabilitation in children and adolescents with somatic conditions is inherently difficult and limited by ethical constraints. Nevertheless, using controlled research designs, researchers should prioritize uncovering which factors influence the effectiveness of rehabilitation programs and which outcomes are affected. This is indispensable to build a strong knowledge base, formulate specific guidelines, and firmly establish psychosocial rehabilitation in children and adolescents with somatic conditions as a distinct scientific field. In this paper, we focused specifically on somatic conditions as a first step toward understanding the potential impact of psychosocial rehabilitation in pediatric populations. However, future research should expand the scope to include other medical indications where psychosocial factors are critical, such as chronic illness management, neurological disorders, and conditions with significant psychological comorbidity. By broadening the focus, we can explore the full potential of psychosocial rehabilitation to support overall well-being and improve health outcomes across a wider range of health conditions.

## Data Availability

The original contributions presented in the study are included in the article/Supplementary Material, further inquiries can be directed to the corresponding author.
