# Supplementary material for: Taking stock of psychosocial rehabilitation in children and adolescents: a systematic review with meta-analysis
Source: Front Rehabil Sci. 2025 Nov 26;6:1568727. doi: 10.3389/fresc.2025.1568727 (PMC12689517; doi:10.3389/fresc.2025.1568727)
Supplement: Supplementary file 2 [file Table2.docx]

**Supplementary Materials**

Supplementary Section 1: Manual for primary study quality rating 2

Supplementary Table 1: Primary study quality rating 4

Supplementary Section 2: Details on effect size calculations and data analysis 5

Supplementary Section 3: Simplified analysis code 6

References 14

# Supplementary Section 1: Manual for primary study quality rating

***Newcastle-Ottawa Scale adapted for cross-sectional studies***

Reference: Herzog R, Álvarez-Pasquin MJ, Díaz C, Del Barrio JL, Estrada JM, Gil Á. Are healthcare workers’ intentions to vaccinate related to their knowledge, beliefs and attitudes? A systematic review. BMC Public Health 2013; 13(1): 154. DOI: 10.1186/1471-2458-13-154

**Selection:** (Maximum 5 stars)

1) Representativeness of the sample:

a) Truly representative of the average in the target population. * (all subjects or random sampling)

b) Somewhat representative of the average in the target population. * (non-random sampling)

c) Selected group of users.

d) No description of the sampling strategy.

2) Sample size:

a) Justified and satisfactory. *

b) Not justified.

3) Non-respondents:

a) Comparability between respondents and non-respondents characteristics is established, and the response rate is satisfactory. *

b) The response rate is unsatisfactory, or the comparability between respondents and non-respondents is unsatisfactory.

c) No description of the response rate or the characteristics of the responders and the non-responders.

4) Ascertainment of the exposure (risk factor):

a) Validated measurement tool. **

b) Non-validated measurement tool, but the tool is available or described.*

c) No description of the measurement tool.

**Comparability:** (Maximum 2 stars)

1) The subjects in different outcome groups are comparable, based on the study design or analysis. Confounding factors are controlled.

a) The study controls for the most important factor (select one). *

b) The study control for any additional factor. *

**Outcome:** (Maximum 3 stars)

1) Assessment of the outcome:

a) Independent blind assessment. **

b) Record linkage. **

c) Self report. *

d) No description.

2) Statistical test:

a) The statistical test used to analyze the data is clearly described and appropriate, and the measurement of the association is presented, including confidence intervals and the probability level (p value). *

b) The statistical test is not appropriate, not described or incomplete.

This scale has been adapted from the Newcastle-Ottawa Quality Assessment Scale for cohort studies to perform a quality assessment of cross-sectional studies for the systematic review, “Are Healthcare Workers’ Intentions to Vaccinate Related to their Knowledge, Beliefs and Attitudes? A Systematic Review”.

We have not selected one factor that is the most important for comparability, because the variables are not the same in each study. Thus, the principal factor should be identified for each study.

In our scale, we have specifically assigned one star for self-reported outcomes, because our study measures the intention to vaccinate. Two stars are given to the studies that assess the outcome with independent blind observers or with vaccination records, because these methods measure the practice of vaccination, which is the result of true intention.

# Supplementary Table 1: Primary study quality rating

|  | **Selection** | | | |  | **Comparability** |  | **Outcome** | |  |  |  |  |  |
| --- | --- | --- | --- | --- | --- | --- | --- | --- | --- | --- | --- | --- | --- | --- |
| **Short reference** | **1.** | **2.** | **3.** | **4.** |  | **1.** |  | **1.** | **2.** |  | **Selection total** | **Comparability total** | **Outcome total** | **Overall score** |
| Banez et al . (2014) | 1 | 0 | 0 | 2 |  | 1 |  | 1 | 1 |  | 3 | 1 | 2 | 6 |
| **Benore et al. (2015)** | **1** | **0** | **1** | **2** |  | **1** |  | **1** | **1** |  | **4** | **1** | **2** | **7** |
| **Däggelmann et al. (2017)** | **1** | **0** | **0** | **2** |  | **1** |  | **1** | **1** |  | **3** | **1** | **2** | **6** |
| Ennis et al. (2014) | 1 | 0 | 0 | 2 |  | 1 |  | 1 | 1 |  | 3 | 1 | 2 | 6 |
| Goldbeck et al. (2011) | 1 | 1 | 1 | 2 |  | 1 |  | 1 | 1 |  | 5 | 1 | 2 | 8 |
| Hampel et al. (2020) | 0 | 0 | 0 | 2 |  | 1 |  | 1 | 1 |  | 2 | 1 | 2 | 5 |
| Kasia et al. (2021) | 1 | 1 | 0 | 1 |  | 1 |  | 1 | 1 |  | 3 | 1 | 2 | 6 |
| **Maynard et al. (2010)** | **1** | **0** | **0** | **1** |  | **1** |  | **1** | **1** |  | **2** | **1** | **2** | **5** |
| Nelson et al. (2019) | 1 | 1 | 0 | 2 |  | 1 |  | 1 | 1 |  | 4 | 1 | 2 | 7 |
| Pakhomova et al. (2022) | 1 | 0 | 0 | 0 |  | 1 |  | 1 | 1 |  | 1 | 1 | 2 | 4 |
| Papp et al. (2021) | 0 | 0 | 0 | 1 |  | 0 |  | 1 | 1 |  | 1 | 0 | 2 | 3 |
| **Riedl et al. (2022)** | **1** | **1** | **0** | **2** |  | **1** |  | **1** | **1** |  | **4** | **1** | **2** | **7** |
| Risko (2019) | 1 | 1 | 0 | 2 |  | 1 |  | 1 | 1 |  | 4 | 1 | 2 | 7 |
| Schiel et al. (2017) | 1 | 1 | 0 | 2 |  | 1 |  | 1 | 1 |  | 4 | 1 | 2 | 7 |
| Singer and Drotar (1989) | 0 | 0 | 0 | 1 |  | 1 |  | 0 | 0 |  | 1 | 1 | 0 | 2 |
| Svatenkova (2016) | 1 | 0 | 0 | 1 |  | 1 |  | 1 | 0 |  | 2 | 1 | 1 | 4 |
| Xodo (2020) | 0 | 0 | 0 | 0 |  | 1 |  | 0 | 0 |  | 0 | 1 | 0 | 1 |
| Zakrepina et al. (2020) | 1 | 0 | 0 | 2 |  | 1 |  | 1 | 1 |  | 3 | 1 | 2 | 6 |

Studies set in boldface were included in our meta-analytic review.

# Supplementary Section 2: Details on effect size calculations and data analysis

*Cohen d from means and standard deviations (Borenstein et al., 2021)*

$$\mathrm{Cohen}d=\frac{M_{control}- M_{disease}}{\sqrt{\frac{{(n}_{control}-1) \times{SD}_{control}^{2} + {(n}_{disease}-1) \times{SD}_{disease}^{2}}{n_{control}+ n_{disease} - 2}}}$$

$${Variance}_{d}=\frac{n_{control}+ n_{disease}}{n_{control} \times n_{disease}}+ \frac{d^{2}}{2 \times(n_{control}+ n_{disease})}$$

*Cohen d from correlation coefficient (Borenstein et al., 2021)*

$$Cohen d=\frac{2 \times r}{\sqrt{1-r^{2}}}$$

$${Variance}_{d}=\frac{4 \times{Variance}_{r}}{{(1-r^{2})}^{3}}$$

$${Variance}_{r}\frac{({1- r^{2})}^{2}}{n-1}$$

$${{Variance}_{OR}=e}^{{Variance}_{logOR}}$$

# Supplementary Section 3: Simplified analysis code

# Before running the script, please download the dataset at the following URL:

# <https://osf.io/tf9zj/?view_only=005c9a6a90384fce8be44b4c74928644>

############# Libraries #############

library(data.table)

library(readxl)

library(ggplot2)

library(metafor)

library(car)

library(ggplot2)

library(metaviz)

library(robumeta)

library(ggtext)

library(cowplot)

library(dplyr)

############# Functions #############

# Improved round function

round_2 <- function(x, digits = 2, return.numeric = FALSE){

if(is.numeric(x) == FALSE) {

temp <- as.numeric(as.character(x))

}

if(is.numeric(x) == TRUE) {

temp <- x

}

temp <- sprintf(paste0("%.", digits, "f"), temp)

temp <- trimws(temp, which = "both")

if(return.numeric == TRUE) {

temp <- as.numeric(as.character(temp))

}

return(temp)

}

# Function that rounds a value to a defined number of digits and removes the leading zero (e.g., for r, p, etc.)

format_p <- function(x, digits=3){

formatting_expression <- paste0("%.", digits, "f")

formatted_value <- sub("^(-?)0.", "\\1.", sprintf(formatting_expression, x))

return(formatted_value)

}

# Function for rem tables

rma.mv_table <- function(rma.mv_object, model_name = NA){

name <- model_name

predictor <- rownames(rma.mv_object$beta)

k <- rma.mv_object$k

estimate <- as.numeric(rma.mv_object$beta)

ll <- rma.mv_object$ci.lb

ul <- rma.mv_object$ci.ub

sigSymbols <-

symnum(

rma.mv_object$pval,

na = FALSE,

cutpoints = c(0, 0.001, 0.01, 0.05, 1),

symbols = c("***", "**", "*", ""),

legend=FALSE)

estimate_ci <-

paste0(

round_2(estimate),

sigSymbols,

" [",

round_2(ll),

"; ",

round_2(ul),

"]")

pval <- rma.mv_object$pval

W <- diag(1/rma.mv_object$vi)

X <- model.matrix(rma.mv_object)

P <- W - W %*% X %*% solve(t(X) %*% W %*% X) %*% t(X) %*% W

i2 <- round_2(100 * sum(rma.mv_object$sigma2) / (sum(rma.mv_object$sigma2) + (rma.mv_object$k-rma.mv_object$p)/sum(diag(P))))

v_between <-

paste0(

ifelse(round_2(rma.mv_object$sigma2[1]) < 0.01, "< 0.01", round_2(rma.mv_object$sigma2[1])),

" (",

round_2((100 * rma.mv_object$sigma2 / (sum(rma.mv_object$sigma2) + (rma.mv_object$k-rma.mv_object$p)/sum(diag(P))))[1]),

"%)")

v_within <-

paste0(

ifelse(round_2(rma.mv_object$sigma2[2]) < 0.01, "< 0.01", round_2(rma.mv_object$sigma2[2])),

" (",

round_2((100 * rma.mv_object$sigma2 / (sum(rma.mv_object$sigma2) + (rma.mv_object$k-rma.mv_object$p)/sum(diag(P))))[2]),

"%)")

temp_table <-

data.table(

model = name,

predictor = predictor,

k = k,

estimate = estimate,

label = estimate_ci,

ll = ll,

ul = ul,

p = pval,

i2 = i2,

v_between = v_between,

v_within = v_within)

return(temp_table)

}

######### Import and data modification #########

# Import MA dataset -- insert path for the directory containing the file dataset.xlsx

screv <-

readxl::read_xlsx(

"your_computer/dataset.xlsx",

col_names = TRUE,

sheet = 1)

# Make new colnames

colnames(screv) <- make.names(colnames(screv))

# Convert dataframe to data.table

screv <- as.data.table(screv)

# Define which columns should be of which class

numeric_columns <-

c(

"Year",

"N.total",

"N.male",

"N.female",

"Mean.age.at.admission",

"M.pre",

"SD.pre",

"M.post",

"SD.post",

"Mean.duration.of.intervention..in.days.",

"Time.of.follow.up..in.days.post.admission.")

factor_columns <-

c(

"Short.reference",

"Control.group",

"Assessment.method")

# Batch class conversion using for loops

for(i in numeric_columns){

screv[[i]] <- as.numeric(as.character(screv[[i]]))

}

for(i in factor_columns){

screv[[i]] <- as.factor(screv[[i]])

}

# Create effect size ID

screv$Effect.size.ID <- 1:nrow(screv)

# Create labels

screv$label <-

c("Benore et al. (2015)/1", "Benore et al. (2015)/2", "Benore et al. (2015)/3", "Benore et al. (2015)/4", "Däggelmann et al. (2017)/5", "Däggelmann et al. (2017)/6", "Däggelmann et al. (2017)/7", "Maynard et al. (2010)/8", "Maynard et al. (2010)/9", "Maynard et al. (2010)/10", NA, "Riedl et al. (2022)/12", NA, NA)

screv$label <- factor(screv$label, levels = screv$label)

screv$label_rev <- factor(screv$label, levels = rev(levels(screv$label)))

# Recode outcome

screv$outcome_recoded <-

dplyr::recode_factor(

screv$Outcomes,

"QOL (psychosocial)" = "QoL",

"QOL (physical)" = "QoL",

"health related QoL" = "QoL",

"anxiety" = "Anxiety",

"depression" = "Depression",

"motor performance" = "Motor performance",

"QOL" = "QoL",

"fatigue" = "Fatigue",

"well-being, activity, and mood" = "Well-being, activity, and mood")

# Define direction of effect (i.e., whether increase indicates improvement)

screv$direction <-

car::recode(

screv$Outcomes,

"

'anxiety' = 'negative';

'depression' = 'negative';

'QOL (psychosocial)' = 'positive';

'QOL (physical)' = 'positive';

'motor performance' = 'positive';

'QOL' = 'positive';

'fatigue' = 'negative';

'Functional status' = 'positive';

'Physical mobility' = 'positive';

'Medication usage' = 'negative';

'well-being, activity, and mood' = 'positive';

'health related QoL' = 'positive' ")

# Compute Cohen d for repeated measures

effsize <-

metafor::escalc(

measure = "SMD",

n1i = screv$N.total,

n2i = screv$N.total,

m1i = screv$M.post,

m2i = screv$M.pre,

sd1i = screv$SD.post,

sd2i = screv$SD.pre,

var.names=c("yi","vi"))

# Append effsizes to data.table

screv <- cbind(screv, effsize)

# Adjust direction of effect according to direction of interpretation (i.e., whether increase indicates improvement)

effsize_direction <- NULL

for(i in screv$Effect.size.ID){

dat_temp <- subset(screv, Effect.size.ID %in% i)

if(dat_temp$direction %in% "negative"){

effsize_temp <- abs(dat_temp$yi)

} else if(dat_temp$direction %in% "positive"){

effsize_temp <- dat_temp$yi

} else if (is.na(dat_temp$direction) == TRUE){

effsize_temp <- NA

}

effsize_direction <- c(effsize_direction, effsize_temp)

rm(dat_temp, effsize_temp)

}

# Append new direction-adjusted effect size column to the dataset

screv <- cbind(screv, effsize_direction)

# Create some reversed variables for ggplot

screv$Short.reference_rev <-

factor(

screv$Short.reference,

levels = rev(levels(screv$Short.reference)))

screv$outcome_recoded_rev <-

factor(

screv$outcome_recoded,

levels = rev(levels(screv$outcome_recoded)))

# Create CI columns

screv$effsize_direction_LL <-

screv$effsize_direction - sqrt(screv$vi)

screv$effsize_direction_UL <-

screv$effsize_direction + sqrt(screv$vi)

# Multilevel meta-analysis, outcome-independent

ma_all <-

rma.mv(

yi = effsize_direction,

V = vi,

random = ~ 1|Short.reference/Effect.size.ID,

data = screv,

slab = Short.reference)

ma_all_table <- rma.mv_table(ma_all, model_name = "Overall model")

# Multilevel meta-analysis, QOL

ma_qol <-

rma.mv(

yi = effsize_direction,

V = vi,

random = ~ 1|Short.reference/Effect.size.ID,

data = subset(screv, outcome_recoded %in% "QoL"),

slab = Short.reference)

ma_qol_table <- rma.mv_table(ma_qol, model_name = "QoL")

######### Publication bias #########

# Multilevel Egger's test following the apporach laid out by Rodgers and Pustejovsky (2021)

egger_multi <-

rma.mv(

yi = effsize_direction,

V = vi,

random = ~ 1|Short.reference/Effect.size.ID,

mods = ~ sqrt(vi),

data = screv)

egger_multi

# Multilevel Egger's test with sandwich estimators following the apporach laid out by Rodgers and Pustejovsky (2021)

egger.sand <-

robu(

formula = effsize_direction ~ 1 + sqrt(vi),

data = screv,

studynum = Short.reference,

var.eff.size = vi)

egger.sand

######### Plots #########

# Default text settings for plots #

text_settings <-

element_text(

colour="black",

size=14,

face="bold")

# Define colors for outcomes

outcome_colors <-

c(

"QoL" = "#A71B4B",

"Anxiety" = "#DE5925",

"Depression" = "#F39B29",

"Motor performance" = "#FBD476",

"Fatigue" = "#FEFD05",

"Well-being, activity, and mood" = "#A5E8AD",

"Functional status" = "#22C4B3",

"Physical mobility" = "#0090B5",

"Medication usage" = "#584B9F")

##### Custom forest plot for overall model using ggplot #

# Define common theme for the polygon boxes below the forest plot

common_theme <- theme(

panel.grid.major.y = element_line(linewidth = 1, linetype = "solid", color = "darkgrey"),

panel.grid.major.x = element_line(linewidth = 0.5, linetype = "dotted", color = "black"),

axis.title.y = element_blank(),

panel.border = element_rect(colour = "black", fill = NA, linewidth = 1),

panel.background = element_rect(fill = "white"),

plot.background = element_rect(fill = "white"),

legend.background = element_rect(fill = "white", colour = "black", linewidth = 0.8),

legend.position = "right",

axis.text.y = text_settings,

axis.text.x = element_text(face = text_settings$face, colour = text_settings$colour, size = text_settings$size, angle = 45, hjust = 1),

axis.title.x = element_markdown(face = text_settings$face, colour = text_settings$colour, size = text_settings$size, angle = 0),

legend.text = text_settings,

legend.title = text_settings,

plot.margin = margin(0, 0, 0, 0, "cm"),

panel.spacing = unit(0, "cm"))

# Draw polygon box

polygon <-

ggplot()+

geom_vline(xintercept = 0, lty = 1, linewidth = 1, color = "black") +

geom_polygon(

data =

data.frame(

x = c(

ma_all_table$ul,

ma_all_table$estimate,

ma_all_table$ll,

ma_all_table$estimate),

y = c(0.5, 0.48, 0.5, 0.52)),

aes(x = x, y = y),

color = "black",

fill = "white",

linewidth = 1,

inherit.aes = FALSE) +

geom_richtext(

inherit.aes = FALSE,

data = tibble(

x = ma_all_table$ul + 0.23,

y = 0.5,

label = paste0("<i>d</i>\u0305 = ", round_2(ma_all_table$estimate, digits = 2))),

aes(

x = x,

y = y,

label = label),

fill = "white",

label.color = "black",

hjust = 0.5,

size = 3,

fontface = "bold")+

geom_richtext(

inherit.aes = FALSE,

data = tibble(

x = ma_all_table$ul + 0.105,

y = 0.5245,

label = "_"),

aes(

x = x,

y = y,

label = label),

fill = NA,

label.color = NA,

hjust = 0.5,

size = 3,

fontface = "bold")+

scale_x_continuous(

breaks = seq(-0.8, 1.6, 0.2),

limits = (c(-0.8, 1.6)),

labels = c(-0.8, -0.6, -0.4, -0.2, 0, 0.2, 0.4, 0.6, 0.8, 1.0, 1.2, 1.4, 1.6)) +

scale_y_continuous(

limits = c(0.44, 0.56),

breaks = 0.5,

labels = "Summary effect") +

theme_classic()+

labs(x = "Cohen *d*", y = NULL, color = "Outcome")+

common_theme

forest_ma_all_2 <-

ggplot(

data = subset(screv, !(screv$yi %in% NA)) ,

aes(x = Short.reference_rev, fill = outcome_recoded_rev)) +

geom_hline(yintercept = 0, lty = 1, linewidth = 1, color = "black") +

geom_linerange(

aes(

ymin = effsize_direction_LL,

ymax = effsize_direction_UL,

color = outcome_recoded_rev),

position = position_dodge(width = 0.3),

lineend = "round",

linewidth = 1.5) +

geom_point(

aes(

y = effsize_direction,

size = N.total,

color = outcome_recoded_rev),

fill = "white",

shape = 21,

position = position_dodge(width = 0.3),

stroke = 1,

show.legend = FALSE)+

scale_y_continuous(

breaks = seq(-0.8, 1.6, 0.2),

limits = (c(-0.8, 1.6)),

labels = c(-0.8, -0.6, -0.4, -0.2, 0, 0.2, 0.4, 0.6, 0.8, 1.0, 1.2, 1.4, 1.6)) +

scale_color_manual(

values = outcome_colors,

name = "Outcome",

breaks = levels(droplevels(screv$outcome_recoded))) +

coord_flip() +

theme_classic()+

labs(x = NULL, y = "Cohen *d*", color = "Outcome")+

common_theme+

theme(

axis.text.x = element_blank(),

axis.ticks.x = element_blank(),

axis.title.x = element_blank())

forest_grid <- cowplot::plot_grid(

forest_ma_all_2,

polygon,

ncol = 1,

align = "v",

rel_heights = c(4, 1.2))

# References

Borenstein, M., Hedges, L. V., Higgins, J. P. T., & Rothstein, H. R. (2021). *Introduction to meta-analysis* (2nd ed.). Wiley.
